# Author Correction: Loss-of-function mutations in PLD4 lead to systemic lupus erythematosus

**DOI:** 10.1038/s41586-025-09692-7

**Published:** 2025-10-10

**Authors:** Qintao Wang, Honghao Zhu, Xiangwei Sun, Changming Zhang, Shuangyue Ma, Ying Jin, Jinjian Fu, Chenlu Liu, Jiahui Peng, Ruoran Wang, Lin Liu, Yi Zeng, Cheng Gong, Qing Zhou, Xiaomin Yu, Zhihong Liu

**Affiliations:** 1 Liangzhu Laboratory, Zhejiang University, Hangzhou, China; 2 National Clinical Research Center of Kidney Diseases, Jinling Hospital, Affiliated Hospital of Medical School, Nanjing University, Nanjing, China; 3 Life Sciences Institute, Zhejiang University, Hangzhou, China; 4 Urology and Nephrology Center, Department of Nephrology, Zhejiang Provincial People’s Hospital, Affiliated People’s Hospital, Hangzhou Medical College, Hangzhou, China; 5 Department of Rheumatology, Sir Run Run Shaw Hospital, Zhejiang University School of Medicine, Hangzhou, China

**Keywords:** Lupus nephritis, Toll-like receptors, Disease genetics

Correction to: *Nature* 10.1038/s41586-025-09513-x Published online 10 September 2025

In the version of this article initially published, due to figure preparation mistakes, the image shown in Fig. 2g for IL-23 was a duplicate of the GM-CSF image below; in Extended Data Fig. 2c, the C1 plot for IFN-α was a duplicate of the C1 plot for IL-1β; and in Extended Data Fig. 2d, the C2 plot was a duplicate of P2. The figures and the source data for Extended Data Fig. 2c have been amended in the HTML and PDF versions of the article.

